# *Announcement*: National Chronic Obstructive Pulmonary Disease Awareness Month — November 2017

**DOI:** 10.15585/mmwr.mm6644a8

**Published:** 2017-11-10

Chronic obstructive pulmonary disease (COPD), which includes emphysema and chronic bronchitis, makes breathing difficult for the 16 million U.S. residents who have received a diagnosis of COPD and millions more who are not aware that they have it (*1*). COPD is the third leading cause of death in the United States (*1*). In collaboration with federal and nonfederal partners, the National Heart, Lung, and Blood Institute (NHLBI) released the COPD National Action Plan in May 2017 (*1*). This document provides a framework for reducing COPD’s impact with roles for advocates and nonprofit organizations, health professionals, researchers, and patients and caregivers.

November is National COPD Awareness Month, an observance supported by NHLBI’s COPD: Learn More, Breathe Better campaign. More information about COPD is available from CDC at https://www.cdc.gov/copd and from NHLBI at https://www.nhlbi.nih.gov/health/educational/copd.
